# Upstream process optimization and micro- and macrocarrier screening for large-scale production of the oncolytic H-1 protoparvovirus

**DOI:** 10.1007/s00253-021-11642-y

**Published:** 2021-11-16

**Authors:** Daniel Wohlfarth, Veronika Frehtman, Marcus Müller, Martin Vogel, Linh Minh Phuc Phan, Adrian Brunecker, Barbara Leuchs

**Affiliations:** German Cancer Research Center, Tumor Virology F010, Im Neuenheimer Feld 280, 69120 Heidelberg, Germany

**Keywords:** Protoparvovirus H-1PV production, Scale-up, Serum-free, Bead-to-bead transfer, Microcarrier, Macrocarrier

## Abstract

**Abstract:**

The oncolytic virus H-1PV is a promising candidate for various cancer treatments. Therefore, production process needs to be optimized and scaled up for future market release. Currently, the virus is produced with minimum essential medium in 10-layer CellSTACK® chambers with limited scalability, requiring a minimum seeding density of 7.9E3 cells/cm^2^. Production also requires a 5% fetal bovine serum (FBS) supplementation and has a virus yield up to 3.1E7 plaque-forming units (PFU)/cm^2^. Using the animal-free cell culture medium VP-SFM™ and a new feeding strategy, we demonstrate a yield boost by a mean of 0.3 log while reducing seeding density to 5.0E3 cells/cm^2^ and cutting FBS supplementation by up to 40% during the production process. Additionally, FBS is completely removed at the time of harvest. Eleven commercial micro- and macrocarriers were screened regarding cell growth, bead-to-bead transfer capability, and virus yield. We present a proof-of-concept study for producing H-1PV on a large scale with the microcarrier Cytodex® 1 in suspension and a macrocarrier for a fixed-bed iCELLis® bioreactor. A carrier-based H-1PV production process combined with an optimized cell culture medium and feeding strategy can facilitate future upscaling to industrial-scale production.

**Key points:**

• *Virus yield increase and FBS-free harvest after switching to cell culture medium VP-SFM™*.

• *We screened carriers for cell growth, bead-to-bead transfer capability, and H-1PV yield*.

• *High virus yield is achieved with Cytodex® 1 and macrocarrier for iCellis® in Erlenmeyer flasks*.

**Supplementary Information:**

The online version contains supplementary material available at 10.1007/s00253-021-11642-y.

## Introduction

According to the World Health Organization (WHO), cancer was the second leading cause of death worldwide in 2018, with an estimated 9.6 million deaths. The economic damage associated with cancer is significant and increasing, totaling approximately $1.16 trillion in 2010 alone (Stewart and Wild, 2014). Oncolytic virus (OV) therapy represents a promising approach to treating this disease. OVs are genetically engineered or naturally occurring viruses that selectively destroy cancer cells without harming healthy tissue (Fukuhara et al., 2016). In 2015, the first OV therapeutic (T-VEC or Imlygic™) was approved by the Food and Drug Administration (FDA) (https://www.fda.gov/vaccines-blood-biologics/cellular-gene-therapy-products/imlygic-talimogene-laherparepvec) and followed by the European Medicines Agency (EMA) (https://www.ema.europa.eu/en/medicines/human/EPAR/imlygic). Other OV therapeutics based on different virus platforms (https://webs.iiitd.edu.in/raghava/ovirustdb/clinical.php) are also in the development pipeline.

The OV drug ParvOryx utilizes the wild-type parvovirus H-1PV, which belongs to the genus *Protoparvovirus* (Cotmore et al., 2014). It demonstrated oncolytic and oncosuppressive properties during preclinical proof-of-concept studies in various cultured cell lines, and in animal (Rommelaere et al., 2010; Nuesch et al., 2012) and xenograft models against several human tumor species (Geletneky et al., 2010; Faisst et al., 1998; Angelova et al., 2009a,b; Dupressoir et al., 1989). H-1PV also showed safety and immunogenic activity in clinical phase I/IIa studies (Geletneky et al., 2012, Geletneky et al., 2017) and phase II studies (Hajda et al., 2021). Considering a market release of H-1PV in the future, the production capacity must be increased and the process optimized.

In general, an ideal bioprocess for producing biologics uses a suspension cell line to facilitate scalability and chemically defined, animal component-free cell culture media to avoid challenges concerning lot-to-lot variation, animal welfare, supply, cost, and potential regulatory restrictions in the future. However, the scalability of the established H-1PV production process is limited because it uses an adherent human cell line, with most of the generated virus being cell-associated at harvest. Additionally, little effort has been invested to render the process free of animal components. The currently employed process was established with minimum essential medium with 5% fetal bovine serum (FBS) in stationary culture using 10-layer CellSTACK® chambers (Corning, Kennebunk, USA). We were able to significantly reduce the amount of FBS needed, ultimately resulting in FBS-free virus harvest and a higher yield, by adapting and optimizing the culture medium VP-SFM™ with a new feeding strategy. Additionally, we conducted a scale-up proof-of-concept study to produce H-1PV with different types of microcarriers and macrocarriers for respective use in suspension or a fixed bed. Highest virus yield was achieved with Cytodex® 1 (Cytiva, Uppsala, Sweden) and macrocarrier from iCELLis® (Pall, Hoegaarden, Belgium). The application of VP-SFM™ in combination with the demonstrated feeding strategy was tested in iCELLis® nano benchtop bioreactor for upscaling production to iCELLis® 500.

## Material and methods

### Cell line and cell culture media

NB-324 K human newborn kidney cells (Tattersall and Bratton, 1983) transformed with simian virus 40 were cultured at 37 °C either in minimum essential medium (MEM, Sigma, Steinheim am Albuch, Germany) with 0% or 5% heat-inactivated fetal bovine serum (FBS, Biowest, Nuaille, France) or VP-SFM™ medium (Thermo Fisher, New York, USA) with 0%, 1%, 2%, or 5% FBS in a 5% CO_2_ atmosphere. The cells were previously negatively tested for mycoplasma (Multiplexion GmbH, Heidelberg, Germany). Cell culture media were supplemented with 100 U/ml penicillin, 100 µg/ml streptomycin, and 2 mM or 5 mM l-glutamine (Thermo Fisher, New York, USA) for MEM and 4 mM or 6 mM l-glutamine for VP-SFM™, respectively (Supplemental Table S1).

### Direct and indirect cell counting and cell growth assay

For cell counting with trypan blue, the cell culture medium was removed, and the cell layer or carriers were washed with phosphate-buffered saline (PBS), trypsinated with approximately 0.017 ml/cm^2^ PBS/1 mM ethylenediaminetetraacetic acid (EDTA) 0.25% trypsin (Gibco, Grand Island, USA) at 37 °C until full cell detachment was observed by microscope. Trypsination was stopped with 5% FBS-supplemented cell culture medium. Then, 5 µl of cell suspension was mixed with an equal volume of 0.4% trypan blue staining solution (Invitrogen, Carlsbad, USA) and transferred onto Countess chamber slides (Invitrogen, Carlsbad, USA) to count the cells in the Countess cell counter (Invitrogen, Carlsbad, USA).

Citric-crystal violet staining was performed to count cell nuclei on carriers to determine multiplicity of infection (MOI). Here, 1 ml of evenly suspended culture with carriers was removed and allowed to settle for 15 min. The supernatant was discarded. The carriers with cells were washed with PBS once, then resuspended in 0.1 M citric acid, containing 0.1% (w/v) crystal violet and incubated at 37 °C for 1 h. The mixture was then gently pipetted and the cell number was determined by counting the released nuclei using a Neubauer cell counting chamber.

The glucose concentration was measured with a glucose blood sugar measuring device (STADA, Bad Vilbel, Germany). For this, one drop of cell culture medium was placed on the glucose test strip and measured with the device. The measured glucose value is expressed in mg/dl. After unit conversion into mmol/l, the total consumed glucose was calculated.

### Virus stock, time of infection, and quantification

For H-1PV production, a virus stock of wild-type H-1PV was produced via transfection (Kestler et al., 1999) of NB-324 K cells and subsequently propagated by two rounds of infection. The virus was quantified with a plaque formation assay (see Leuchs et al., 2016), for a description of the method). Time of infection (TOI) was either at seeding, based on Countess cell count (simultaneous infection and seeding), or at day 3 (non-simultaneous infection), based on citric acid nuclei count for carrier cultures or Countess cell count for stationary cultures.

### Cell cultivation and virus production in stationary culture

#### Simultaneous seeding and infection

3.6E4 cells/cm^2^ were seeded in T175 flasks with 20 ml of either MEM full or VP-SFM™ full, VP-SFM™ w/o FBS, or VP-SFM™ full Gln (see Supplemental Table S1) and simultaneously infected. Cells were harvested at 4 days postinfection (dpi).

#### Non-simultaneous seeding and infection

7.9E3 cells/cm^2^ were seeded in T175 flask with 20 ml of either MEM full, VP-SFM™ full, or VP-SFM™ cell expansion medium (Supplemental Table S1). After 3 days of cell expansion, the medium was completely exchanged with MEM full for cells that were seeded in MEM or for cells seeded in VP-SFM™ with VP-SFM™ w/o FBS or VP-SFM™ infection medium. Simultaneously, cells were infected with a MOI of 0.01 or 0.05 according to the Countess cell count of a reference T175 flask. On day 5, another 100% medium exchange with VP-SFM™ w/o FBS was performed for cells that had been in VP-SFM™ infection medium since day 3 (Supplemental Fig. S1). Cells were harvested on day 7.

#### Carrier preparation

Microcarrier and macrocarrier (termed “carriers” when both systems are discussed) characterization is shown in Table 1. Microcarriers were handled and stored in bottles that were siliconized with Sigmacote (Sigma-Aldrich, Steinheim am Albuch, Germany) according to the manufacturer’s instructions. Non-cationic microcarriers were hydrated and autoclaved in aqua ad. injectable (B. Braun, Melsungen, Germany), cationic microcarriers in 1 × PBS without Ca^2+^ and Mg^2+^, according to the manufacturer’s instructions. Macrocarriers were sterile when supplied and hydrated in cell culture medium for 30 min at 37 °C before use.

**Table 1:** Overview of microcarriers and macrocarriers

Carrier	Brand	Name	Abbreviation	Charge	Structure
Microcarrier for suspension	Pall (Hoegaarden, Belgium)	SoloHill® Hillex®II	HII	Cationic (DEAE group)	High-density, solid
		SoloHill® Star-Plus	SP	Neutral	Solid
		SoloHill® Plastic	P	Neutral	Solid
		SoloHill® Plastic Plus	PP	Cationic (DEAE group)	Solid
	Corning (Kennebunk, USA)	Enhanced attachment CellBIND®	EA	Neutral	Solid
		Low Concentration Synthemax® II	SII	RGD* modified	Solid
	Cytiva (Uppsala, Sweden)	Cytodex® 1	CD1	Cationic (DEAE group)	Solid
		Cytopore™ 1	CP1	Cationic (DEAE group)	Porous
		Cytopore™ 2	CP2	Cationic (DEAE group)	Porous
Macrocarrier for fixed-bed	Eppendorf (Enfield, USA)	Fibra-Cel®	FC	Neutral	Macroporous
	Pall (Hoegaarden, Belgium)	Macrocarrier from iCELLis®	iC	Neutral	Macroporous

^*^RGD-containing sequence from the human ECM protein vitronectin, KGGPQVTRGDVFTMP, which promotes adhesion in a variety of cells

### Cell cultivation and production systems for carriers

#### Screening in 24-well plates

Screening experiments of the carriers were performed in 24-well, ultra-low attachment plates (Corning, Kennebunk, USA) with 1 ml VP-SFM™ full per well, at 37 °C, 5% CO_2_, and 100 rpm orbital agitation with Max Q 2000 CO_2_ Plus (Thermo Fisher Scientific, New York, USA). Stationary controls were treated like carrier samples but seeded in 6-well plates (9.6 cm^2^ growth area) with 2 ml cell culture medium per well without agitation.

For simultaneous seeding and infection, 10 cm^2^ or 11.3 cm^2^ of carrier was added per well to three wells. Macrocarriers from iCELLis® were cut in half to fit into a well. Then, 1 ml VP-SFM™ full with NB-324 K cells, corresponding to a seeding density of 4E4 cells/cm^2^, was added to each well and infected with MOI of 0.01. Cells were harvested 4 days postinfection.

For non-simultaneous seeding and infection, 5 cm^2^ of growth area was added per well in 2 wells. Then, 1 ml VP-SFM™ full with NB-324 K cells, corresponding to a seeding density of 2E4 or 4E4 cells/cm^2^, was added to each well. On day 3, both wells per carrier were pooled and the nuclei from a sample counted. Cells were infected (MOI of 0.01) by adding fresh cell culture medium VP-SFM™ full, including the virus and fresh carrier, doubling the total growth area from 10 to 20 cm^2^ and the cell culture volume from 2 to 4 ml for each pool. The pool of spent and fresh carrier was then split into 3 wells with a 5-cm^2^ growth area and 1 ml cell culture medium per well. Cells were harvested 4 days postinfection on day 7.

#### Microcarriers in an Erlenmeyer flask

After screening, enhanced attachment (EA) and Cytodex® 1 (CD1) microcarriers were selected for upscaling experiments in a 125-ml Erlenmeyer flask with 40 ml VP-SFM™ full and 10 cm^2^/ml growth area, at 37 °C, 5% CO_2_, and 60–70 rpm orbital agitation with Max Q 2000 CO_2_ Plus (Thermo Fisher Scientific, New York, USA). Here, 2E4 cells/cm^2^ was seeded, and agitation was reduced to 0 rpm for 30 min or to 30 rpm for 3 h to promote cell attachment. On day 3, a sample was taken to determine cell density with nuclei count for virus infection (MOI of 0.01), and the virus was added during a 50% medium exchange on the same day with fresh VP-SFM™ full.

#### Virus production in Erlenmeyer flasks with macrocarriers

Fibra-Cel® and macrocarrier from iCELLis® were also tested in 125-ml Erlenmeyer flasks with parameters similar to those described for the microcarriers. However, orbital agitation was 30–100 rpm, and agitation during seeding was either 100 rpm or a cycle of 40 rpm for 1 min and then 0 rpm for 30 min, which was repeated four times to a total seeding time of 2 h.

#### Virus production in Spinner flasks with carriers

EA and CD1 microcarriers were further scaled up in a 250-ml Spinner flask (Integra Biosciences, Switzerland) and Fibra-Cel® and macrocarrier from iCELLis® in a 500-ml Spinner flask (Integra Biosciences, Biebertal, Germany) with 100 ml VP-SFM™ full and 10 cm^2^/ml growth area, at 37 °C, 5% CO_2_, and 15–30 rpm agitation. Then, 2E4 cells/cm^2^ was seeded, and agitation was reduced to 0 rpm for 30 min or a cycle of 40 rpm for 1 min and then 0 rpm for 30 min, which was repeated four times to a total seeding time of 2 h. On day 3, a sample was taken to determine cell density with nuclei count for virus infection (MOI of 0.01 or 0.05), and the virus was added during a 50% medium exchange with VP-SFM™ full on the same day. Cells were harvested 4 days postinfection on day 7.

### Virus production in iCELLis® nano bioreactor

The iCELLis® nano bioreactor system (Pall, Port Washington, USA) was tested in 0.53-m^2^ and 4-m^2^ fixed-bed sizes (Pall, Port Washington, USA). After preparing the fixed-bed according to the manufacturer’s instructions, bioreactors were filled with 850 ml VP-SFM™ cell expansion medium (0.16 ml/cm^2^). For the 4-m^2^ fixed bed, a recirculation loop (Pall, Port Washington, USA) supplying an additional 3150 ml VP-SFM™ cell expansion medium was connected (0.10 ml/cm^2^). Then, 5E3 cells/cm^2^ was seeded for the 0.53-m^2^ fixed-bed or 9E3 cells/cm^2^ for the 4-m^2^ fixed-bed and maintained at 37 °C, pH 7.3, above 30–40% dissolved oxygen. After 3 days of cell expansion, several macrocarriers were taken from the top of the fixed bed and cells were counted, followed by infection with an MOI of 0.01 during a 100% medium exchange to VP-SFM™ infection medium. Two days postinfection, an additional 100% medium exchange to VP-SFM™ w/o FBS medium was performed. Cells were harvested 4 days postinfection on day 7.

### Harvest

For carrier cultures in wells, or Erlenmeyer or Spinner flasks, the cell culture medium was removed 4 days postinfection, and then carriers were treated for 30 min at 37 °C with 0.02 ml/cm^2^ 1% Triton® X-100 (Sigma-Aldrich, St. Louis, USA), 0.1 M Tris–HCl, pH 9.5, for cell lysis.

For iCELLis® nano-cultures, the cell culture medium was removed 4 days postinfection. Then, the cells in the fixed bed were rinsed with PBS and lysed with 0.094 ml/cm^2^ (0.53 m^2^) or 0.014 ml/cm^2^ (4 m^2^) detergent-based buffer.

For stationary cultures, cell lysis via a freeze/thaw process was performed. The medium was removed, and infected cells were washed with 1 × PBS. The medium supernatant and detached cells were centrifuged for 5 min at 5000 × *g*. The pellet was washed with PBS, resuspended with 0.02 ml/cm^2^ 0.05 M Tris–HCl, pH 8.7 (VT), for 30 min at 37 °C, and subjected to three freeze (liquid nitrogen) and thaw (37 °C) cycles. In addition, the following steps were taken during medium optimization and FBS reduction: After centrifugation for 5 min at 5000 × *g*, cell debris was discarded. The cell lysate was then sonicated at 48 W for 1 min with a Sonorex Super 10 P ultrasonic homogenizer (Bandelin, Berlin, Germany) and treated with DNAse (50 U/ml, Sigma-Aldrich, Steinheim am Albuch, Germany) after adding 5 mM MgCl_2_ for 30 min at 37 °C to eliminate non-encapsidated viral DNA and contaminating host cell DNA.

## Results

**Fig. 1:**
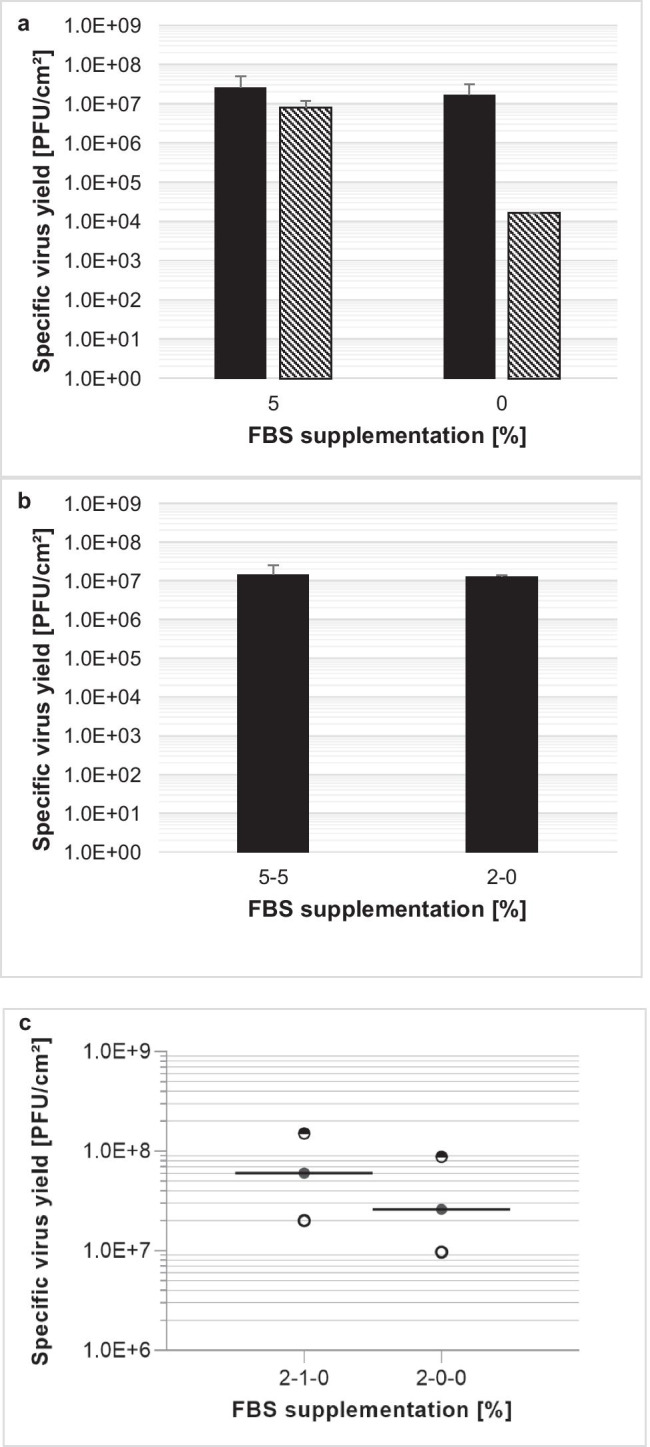
**a** Effect of FBS removal on H-1PV-specific virus yield with MEM (hatching lines) or VP-SFM™ (black) cell culture media. Here, 2.6E4 NB-324 K cells/cm^2^ was simultaneously seeded and infected with a MOI of 0.05. Cells were harvested and lysed 4 days postinfection with a freeze–thaw process in lysis buffer. **b** Similar H-1PV-specific virus yield with 5% FBS or two-step FBS reduction scheme. Here, 3.6E4 NB-324 K cells/cm^2^ was seeded in VP-SFM™ medium supplemented with 5% or 2% FBS. After 3 days of cell expansion, the infection with a MOI of 0.05 and a 100% medium exchange with 5% or 0% FBS was performed. Cells were harvested and lysed 4 days postinfection with a freeze–thaw process in lysis buffer. **c** Boost of H-1PV-specific virus yield with three-step FBS reduction using VP-SFM™. Here, 5E3 NB-324 K cells/cm^2^ was seeded with 2% FBS. After 3 days of cell expansion, the first 100% medium exchange to either 1% or 0% FBS and infection with a MOI of 0.01 was performed for production phase I from day 3 to day 5. Two days postinfection, a second 100% medium exchange was performed without FBS for production phase II: day 5–day 7. Cells were harvested and lysed 4 days postinfection on day 7

**Fig. 2:**
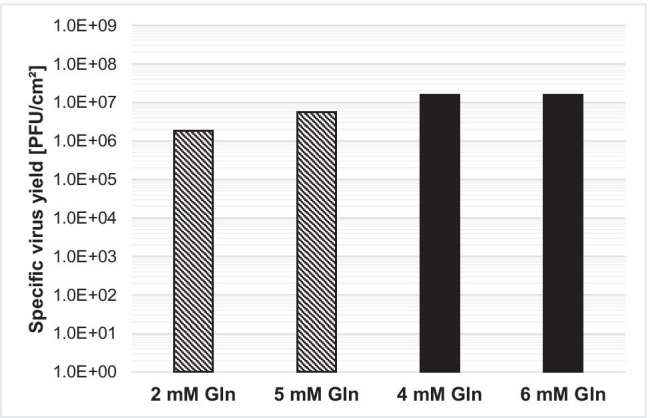
Effect of glutamine supplementation on H-1PV-specific virus yield MEM (hatching lines) or VP-SFM™ (black) medium. Here, 3.6E4 NB-324 K cells/cm^2^ were simultaneously seeded and infected with a MOI of 0.05 in 5% FBS. Cells were harvested and lysed 4 days postinfection

To increase the yield, while lowering production costs and avoiding undesired products of animal origin, VP-SFM™ cell culture medium was tested and compared with a previously described MEM medium (Leuchs et al., 2016), employing simultaneous or non-simultaneous infection and seeding. With simultaneous seeding and infection, omitting FBS from MEM decreased the virus yield from 8.3E6 plaque-forming units (PFU)/cm^2^ to 1.7E4 PFU/cm^2^ (Fig. 1a). VP-SFM™ supplemented with 5% FBS or 0% FBS achieved an increased virus yield compared to MEM, at 2.7E7 PFU/cm^2^ and 1.8E7 PFU/cm^2^, respectively. Glutamine was fully consumed in MEM by the end of the process (data not shown). Therefore, higher glutamine (6 mM for VP-SFM™ and 5 mM for MEM) concentrations were supplemented with 5% FBS for both cell culture media. These conditions only increased the virus yield for MEM from 1.8E6 to 5.6E6 PFU/cm^2^. Nevertheless, VP-SFM™ demonstrated an overall better yield of 1.6E7 PFU/cm^2^ (Fig. 2). Comparing the two cell culture media suggests that 4 mM glutamine supplementation is sufficient for VP-SFM™ and results in higher yields even at lower amounts of FBS than for MEM. Thus, MEM was replaced with VP-SFM™ for further optimizing the process.

Afterward, we compared a two-step with a three-step FBS reduction strategy using 100% medium exchanges. See Fig. S1 for an overview of all medium exchange strategies with VP-SFM™ medium. All strategies started with 5% FBS in the seed train, followed by 3 days of cell expansion in 5% or 2% FBS and a 100% medium exchange with simultaneous infection on day 3 after seeding. In the two-step process, when infection is done with 0% FBS (2–0%) the resulting virus yield of 1.0E7 PFU/cm^2^ was similar to that for 5% FBS supplementation (5–5%) over the whole process (Fig. 1b). In the three-step strategies, the 100% medium exchange on day 3 after seeding with 2% FBS was supplemented with either 1% FBS (2–1-0%) or without FBS (2–0-0%), followed by a second 100% medium exchange without FBS on day 5 for both. With three independent experiments, we demonstrated that the 2–1-0% FBS strategy resulted in twice the yield of the 2–0-0% FBS strategy up to 7.7E7 PFU/cm^2^ (Fig. 1c). In summary, the results indicate that depletion of FBS over the process is feasible with the highest virus yield achieved by applying the 2–1-0% FBS strategy. However, FBS includes components needed for a high virus yield which cannot be supplied by VP-SFM™ alone.

### Chemical cell lysis needed for large-scale H-1PV harvest

**Fig. 3:**
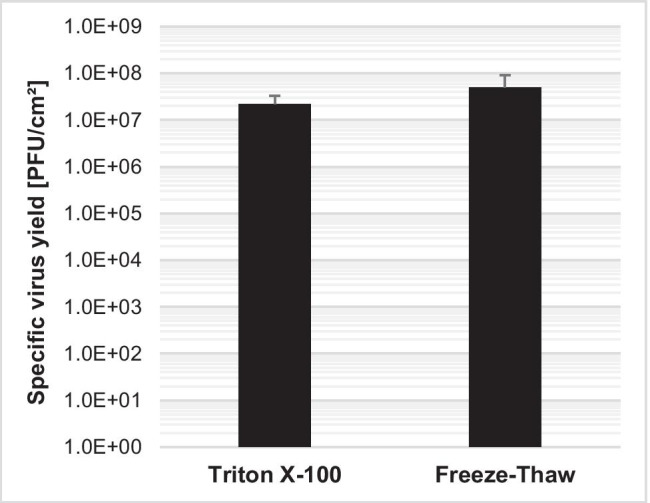
Comparison of harvest with Triton^®^ X-100 process or the freeze–thaw process in VT buffer for cell lysis. NB-324 K cells were seeded in VP-SFM™ with 4E4 cells/cm^2^ in stationary 6-well plates and simultaneously infected with a MOI of 0.01. Cells were harvested and lysed 4 days postinfection

In our process, the majority of infective virus particles are cell-associated at the time of harvest. To harvest H-1PV, a freeze–thaw cell lysis in Tris–EDTA buffer (VTE) (Leuchs et al., 2016) or Tris–HCl buffer (VT) (Leuchs et al., 2017) was previously reported for stationary cultures. For large-scale production with adherent cells on carriers, a scalable cell lysis is required. Therefore, we developed an alternative cell lysis method with Triton® X-100, resulting in a satisfactory virus yield > 2.0E7 PFU/cm^2^ (Fig. 3).

Taken together, medium optimization and the three-step medium exchange strategy with Triton® X-100 lysis constitute a solid basis for upscaling production.

### Screening of cell growth on microcarriers and macrocarriers in a 24-well scale

For large-scale production with adherent cells, microcarriers can be employed for suspension culture or macrocarriers for a fixed-bed bioreactor. We screened cell growth, bead-to-bead-transfer capability, and virus yield for eleven carrier types. Direct cell counting on carriers was difficult. Therefore, we measured glucose consumption as an indicator of growth.

**Fig. 4:**
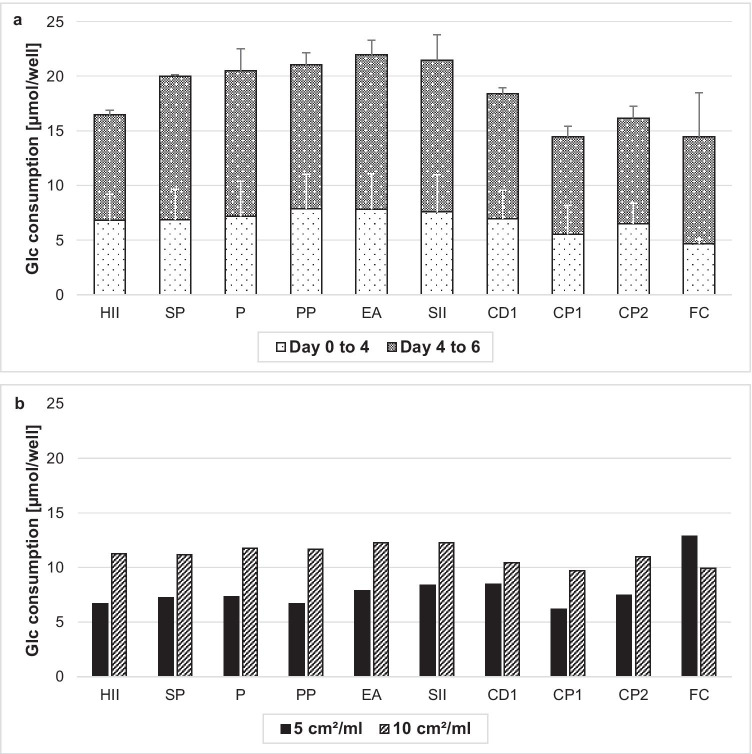
Glucose consumption of NB-324 K cells on different carriers for cell growth screening, shaken at 100 rpm in 24-well plates. **a** Glucose consumption from day 0 to day 4 and day 4 to day 6 with 5 cm^2^/ml and 2E4 seeded cells/cm^2^. **b** Glucose consumption with 4E4 seeded cells/cm^2^ either 5 cm^2^/ml or 10 cm^2^/ml from day 0 to day 4

Figure 4a shows the glucose consumption of cells cultivated on different carriers (without macrocarrier from iCELLis®) for up to 6 days with a surface-to-medium volume ratio of 5 cm^2^/ml and a seeding density of 2E4 cells/cm^2^. All microcarriers show a glucose consumption of 6 to 8 µmol/well within 4 days and 15 to 22 µmol/well after 3 additional days, except for CP1 and FC, which consumed less glucose. In comparison, with doubled seeding cell density of 4E4 cells/cm² and 5 cm^2^/ml medium, glucose consumption was similar to 2E4 seeded cells/cm^2^, while 10 cm^2^/ml showed a 1.5-fold higher glucose consumption within 4 days, except for macrocarrier FC (Fig. 4b). Overall, cell growth was satisfactory at 5 cm^2^/ml and 10 cm^2^/ml for all carriers, with the exception of the porous microcarriers CP1 and CP2.

### Microcarriers are capable of bead-to-bead cell transfer

**Fig. 5:**
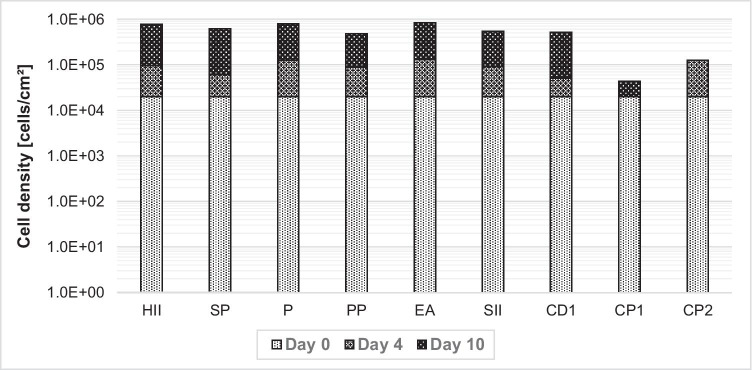
Increase in cell density with bead-to-bead transfer on microcarriers on days 4 and 7. Here, 4E4 NB-324 K cells/cm^2^ and 5 cm^2^/ml were seeded in 24-well plates and shaken at 100 rpm. On days 4 and 7, 50% of medium with microcarriers were taken out and filled up with fresh medium with microcarriers for a constant ratio of microcarriers to medium volume. No increase in cell density of CP1 on day 4 and for CP2 on day 10 was observed

**Fig. 6:**
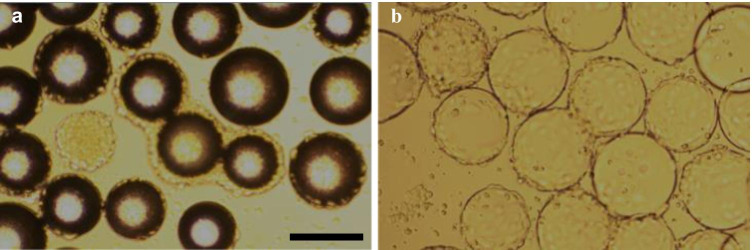
Microscopic observation of cell bridges between confluent and freshly added microcarriers. **a** Enhanced attachment (× 10); **b** Cytodex® 1 (× 10); scale bar = 200 µm

Some cell lines are capable of building individual cell bridges from a confluent microcarrier to a fresh one for continued cell growth. This bead-to-bead transfer without trypsination can facilitate seed train cell expansion because fresh microcarriers only need to be added. To test bead-to-bead transfer capability, cells were seeded on microcarriers, and more microcarriers were added with the fresh cell culture medium by a 1:2 split on day 4 and day 7. Trypan blue cell count of microcarriers after trypsination was performed on day 4 before the 1:2 split and on day 10. According to trypan blue cell count after trypsination, only the non-porous microcarriers were capable of bead-to-bead transfer, as observed by the increase in cells from day 4 to day 10 of up to 5E5 cells/cm^2^ (Fig. 5). In Fig. 6, the bead-to-bead transfer with cell bridges is shown with microcarrier EA and CD1. The capability of bead-to-bead transfer without trypsination suggests good cell expansion capability in scaled-up seed trains for all nonporous microcarriers with NB-324 K cells. This result confirms our findings of a lack of cell growth on porous microcarriers CP1 and CP2.

### Cytodex® 1 and macrocarrier from iCELLis® show the highest H-1PV yield

After characterizing cell growth on the carriers, we applied different production strategies (simultaneous infection/non-simultaneous seeding and infection, with or without bead-to-bead transfer) to identify conditions most suited for high virus production of H-1PV.

**Fig. 7:**
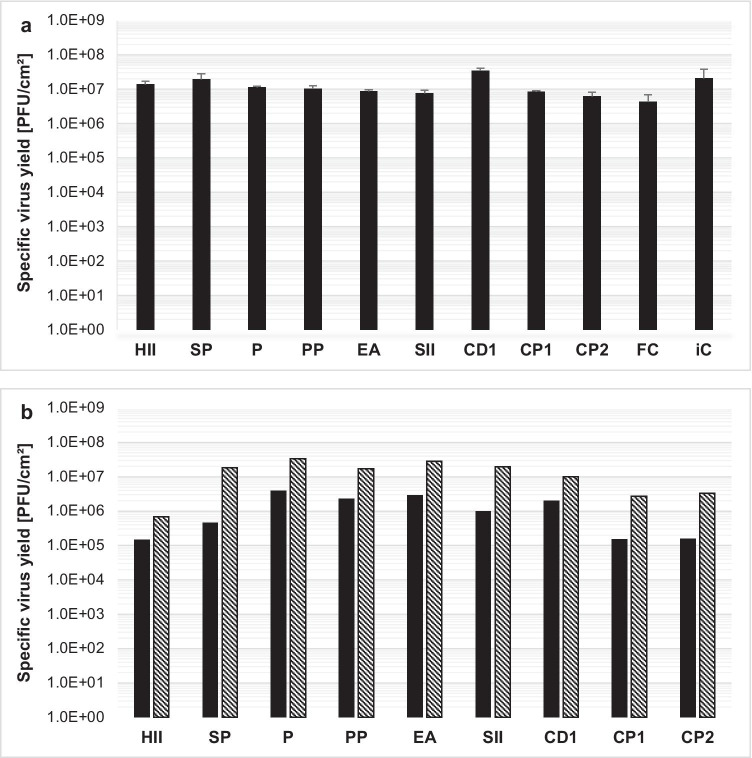
**a** H-1PV-specific virus yield screening with different carriers. Here, 4E4 NB-324 K cells/cm^2^ was seeded with 10 cm^2^/ml (11.2 cm^2^/ml for iC) growth area of each carrier in 24-well plates and shaken at 100 rpm. Cells were harvested and lysed 4 days postinfection. **b** H-1PV-specific virus yield with different microcarriers after bead-to-bead transfer. 2E4 (black) or 4E4 (hatching lines) NB-324 K cells/cm^2^ was seeded with 5 cm^2^/ml growth area of each microcarrier in 24-well plates and orbitally shaken at 100 rpm. After 3 days of cell expansion, cells were infected with a MOI of 0.01, while growth surface and medium volume were doubled by addition of fresh microcarriers in fresh medium, followed by a 1:2 split to return to start conditions of medium volume and microcarrier density. Cells were harvested and lysed 4 days postinfection

Simultaneous infection and seeding of carriers in 24-well, ultra-low attachment plates resulted in similar virus yield for most microcarriers (HII, SP, P, PP, EA, SII, CP1, CP2). The highest yield was achieved with microcarrier CD1 and macrocarrier iC, 3.4E7 PFU/cm^2^, and 2.0E7 PFU/cm^2^, respectively (Fig. 7a). Macrocarrier FC had the lowest yield of 4.2E6 PFU/cm^2^.

For non-simultaneous seeding and infection, in addition to the 4E4 cells/cm^2^ density, a 50% reduced seeding density of 2E4 cells/cm^2^ was tested for higher yield. Three days after seeding, the cells were infected and a 1:2 split, adding fresh cell culture medium and microcarriers to maintain volume and microcarrier density, was performed for bead-to-bead transfer. Virus yield was highest at a seeding density of 4E4 cells/cm^2^, which was similar for most microcarriers compared to simultaneous seeding and infection without bead-to-bead transfer, with the exception of the solid HII and the porous CP1 and CP2 carriers (Fig. 7b). Taken together, high virus production seems possible with all tested strategies but requires a seeding density of 4E4 cells/cm^2^. In addition, HII and porous microcarriers CP1 and CP2 only showed a high yield for simultaneous infection and seeding without bead-to-bead transfer.

**Fig. 8:**
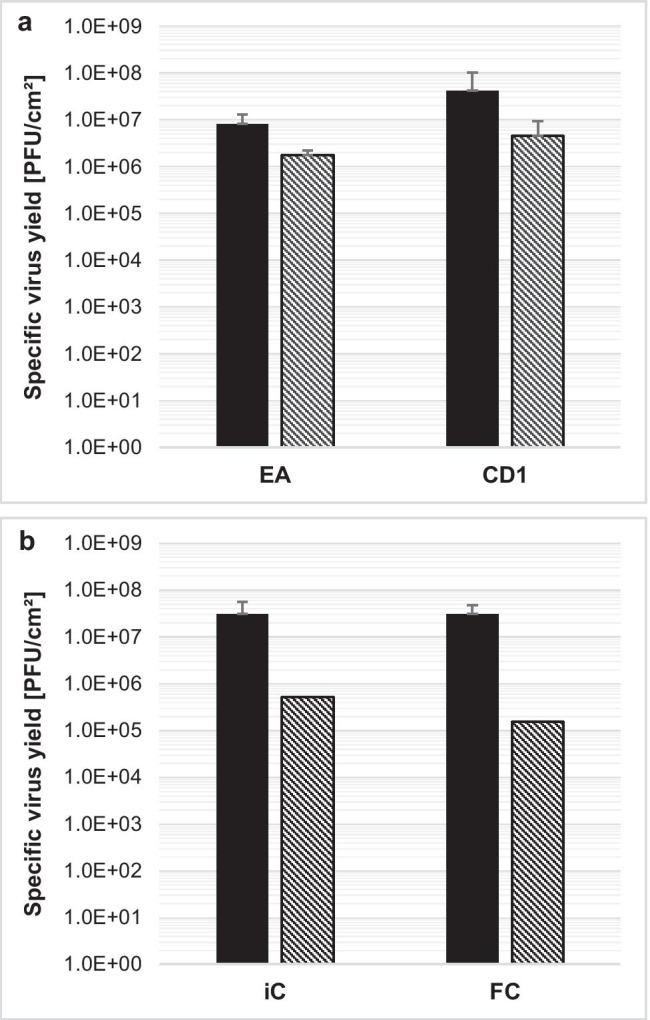
H-1PV-specific virus yield after scale-up of best-performing microcarriers and macrocarriers. Here, 2E4 NB-324 K cells/cm^2^ was seeded with 10 cm^2^/ml growth area (**a** microcarrier EA or CD1; **b** macrocarrier iC or FC) in a VP-SFM™ volume of 40 ml per 125-ml Erlenmeyer flask (black) or 100 ml per Spinner flask (hatching lines). For seeding, a cycle of 1 min at 40 rpm, followed by 30 min at 0 rpm, was repeated four times to a total seeding time of 2 h. Then, agitation was set between 30 and 100 rpm for the Erlenmeyer flask or 40 rpm for the Spinner flask. After 3 days of cell expansion, cells were infected with a MOI of 0.01 and 50% of the medium exchanged with fresh medium. Cells were harvested and lysed 4 days postinfection

Upscaling from 24-well plate format up to 100 ml with Erlenmeyer and Spinner flasks was examined with two selected microcarriers and two macrocarriers. The microcarrier CD1 showed the highest yield, while microcarrier EA surface is comparable to that for 10-layer CellSTACK® chambers. Both macrocarriers for fixed-bed bioreactors were also chosen for upscaling experiments, due to possible limitations associated with 24-well plate, small-scale testing. A wide range of parameters such as seeding and process agitation, carrier densities, cell-seeding densities, MOI, TOI, with/without bead-to-bead transfer, cell culture volume per vessel, and a medium exchange regimen were tested (Supplemental Tables S2 and S3). However, only the most promising parameters with 40 ml cell culture medium in an Erlenmeyer flask and 100 ml in a Spinner flask are shown (Fig. 8a and b).

The microcarrier CD1 reached a yield level of 4.3E7 PFU/cm^2^ in the Erlenmeyer flask, but it was 1 log less when upscaled in the Spinner flask. The microcarrier EA had a lower virus yield than CD1 in all systems.

A 3.0E7 PFU/cm^2^ virus yield was achieved with macrocarrier iC and FC in the Erlenmeyer flask, a macrocarrier density of 10 cm^2^/ml, and a total cell surface of 400 cm^2^. However, when upscaled to the Spinner flask, yield was below 1.0E6 PFU/cm^2^ with 10 cm^2^/ml and 1000-cm^2^ cell surface. The results of CD1 and both macrocarriers in the Erlenmeyer flask confirm that high virus yield is possible in suspension, and these carriers are the best candidates for further upscaling.

### First production in iCELLis® nano bioreactor

Assuming a yield of 3.0E7 PFU/cm^2^ that was generated in the Erlenmeyer flask, with the iCELLis® 500-m^2^ fixed-bed, a batch yield of 1.5E14 PFU can be expected (corresponding to 15,000 doses, each with 1E10 PFU). Therefore, virus production was tested in the downscaled iCELLis® nano system.

**Fig. 9:**
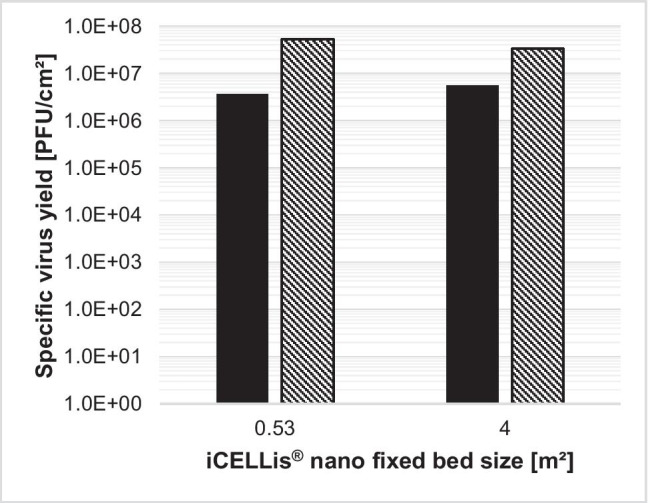
H-1PV-specific virus yield in iCELLis® nano with different fixed-bed and respective Tflask run controls. In separate bioreactor runs, 5E3 cells/cm^2^ (0.53 m^2^) or 9E3 cells/cm^2^ (4 m^2^) was seeded in iCELLis® nano fixed-bed (black) and Tflask run control (hatching lines). After 3 days of cell expansion with VP-SFM™ Cell expansion medium, the first 100% medium exchange to VP-SFM™ Infection medium and infection with a MOI of 0.01 was performed for production phase I from day 3 to day 5. Two days postinfection, a second 100% medium exchange was performed with VP-SFM™ w/o FBS medium for the production phase II: day 5–day 7. Cells were harvested and lysed 4 days postinfection on day 7

For production in iCELLis® nano bioreactor, the newly developed medium exchange strategy 2–1-0% FBS was used. Here, 0.53-m^2^ and 4-m^2^ fixed-bed sizes were tested and resulted in 3.7E6 PFU/cm^2^ and 5.7E6 PFU/cm^2^, respectively (Fig. 9). Both fixed-beds had a comparable but approximately one log step lower yield than did the macrocarrier from iCELLis® in the Erlenmeyer flask and stationary run control.

## Discussion

Patient treatment with the oncolytic parvovirus H-1PV has shown promising results in clinical trials against several types of cancers (Geletneky et al., 2017; Hajda et al., 2021). In preparation for market release, we sought to optimize the culture medium and production process at laboratory scale.

The presented data show the first results for producing H-1PV in a large-scale bioreactor system. However, the process needs to be adapted and optimized according to system parameters such as agitation speed, aeration, seeding cell density, time of infection, multiplicity of infection, and time of harvest.

FBS in cell culture is still common, but problematic in terms of quality, lot-to-lot reproducibility, animal welfare, supply, cost (van der Valk et al., 2018), and potential regulatory restrictions in the future. Therefore, the optimized culture medium VP-SFM™, suitable for FBS-free production (Liu et al., 2017; Rourou et al., 2007; Martinez et al., 2020) and a two-step medium exchange strategy, was adapted to reduce the required amount of FBS by up to 80% for a FBS-free harvest at a comparable virus yield. With an additional medium exchange at 2 dpi and three-step FBS reduction from 2 to 1% and to 0%, a production yield boost of approximately 0.3 log was achieved, while still reducing the FBS needed by up to 40%. By applying this strategy, a high virus production yield with a FBS-free harvest and fewer impurities for the downstream process can be achieved.

In the non-simultaneous seeding and infection process, we could reduce the seeding density from 7.9E3 cells/cm^2^ with MEM-HEPES to 5.0E3 cells/cm^2^ and maintain a similar virus yield after adapting to VP-SFM™. In this way, the cell expansion time could be reduced, and fewer resources were needed for the seed train.

For H-1PV harvest, PBS/EDTA cell detachment and cell lysis in Tris–HCl buffer with successive freeze–thaw cycles have been employed in the past. For production screening on different microcarriers and macrocarriers, a Triton® X-100-based chemical cell lysis was introduced due to the limitations of freeze–thaw lysis for upscaling. With Triton® X-100 lysis, satisfactory production yields were achieved on different microcarriers and macrocarriers. Currently, the detergent Triton® X-100 is considered eco-toxic by regulatory authorities (https://echa.europa.eu/authorisation-list). An alternative, eco-friendlier, and scalable harvest method needs to be developed for future non-experimental applications with a similar or higher efficiency. Tween® 20 (Moleirinho et al., 2018) could be considered an alternative detergent.

To simplify the upscaling process with the anchorage-dependent production cell line, several microcarriers and macrocarriers were tested. After screening in 24-well plates for growth and virus production properties and selected upscaling into Erlenmeyer and Spinner flasks, the solid microcarrier Cytodex® 1 was found to be most suited. Low yield in the Spinner flask was observed with all tested microcarriers and macrocarriers. This was most likely due to device limitations related to propeller shape and speed, resulting in shear stress at the lowest rpm. Indeed, high virus yield has already been reported with Cytodex® 1 for adenoviruses and retroviruses (Wu et al., 2002), vaccinia virus (Liu et al., 2017), and influenza virus A (Tree et al., 2001).

With the exception of porous Cytopore™ 1 and Cytopore™ 2, all other microcarriers showed promising bead-to-bead cell transfer capability during cell growth and virus production, without the need for a trypsination step. If such a regimen can be exploited in larger-scale systems, it could simplify seed train expansion and production scale-up. Furthermore, the porous surface of Cytopore™ 1 and Cytopore™ 2 might reduce the efficacy of cell migration. Additionally, the porous microcarriers showed lower cell growth as indicated by cell count, lower glucose consumption rate, and reduced production yield. Wu et al. (2002) published similar results with human kidney 293 cells. However, in our experiments, the surface pores may have impeded cell detachment after trypsination for cell counting and virus detachment after cell lysis, resulting in lower cell growth and yield. Bead-to-bead transfer was not tested for the macrocarriers because it is not feasible to add more macrocarriers in a fixed-bed bioreactor.

Our experiments in small-scale productions showed promising results with microcarrier Cytodex®1. Therefore, upscaling in a continuous stirred-tank reactor or wave-mixed bioreactor with a cell culture bag seems feasible. Alternatively, the Fibra-Cel® or iCELLis® macrocarriers showed a good production yield in suspension in Erlenmeyer flasks, but it was low in the Spinner flask. However, both macrocarriers are designed for fixed-bed bioreactors, in which a higher yield may be achieved.

Based on the compactness, high virus yield per batch, and ease of scalability of the iCELLis® system, further testing was done in iCELLis® nano bioreactor. The new medium exchange strategy tested in stationary culture was adapted for iCELLis® nano cultivation to reduce FBS content and seeding density. However, virus yield was lower than that in stationary controls and the Erlenmeyer flask.

In summary, with the optimized cell culture medium VP-SFM™ and the new medium exchange strategy, we established a reduction in seeded cell density and FBS, leading to a FBS-free harvest, and tested carriers best suited for a high H-1PV yield, cell growth, and bead-to-bead transfer capability. We demonstrated feasible, carrier-based production and successfully scaled up the process from 24-well plates to Erlenmeyer and Spinner flasks. The combination of carrier cultivation with the new medium exchange strategy was tested in iCELLis® nano bioreactor, but further optimization to increase virus yield is required.

To produce oncolytic H-1PV for future patient application, the process needs to be transferred to a large-scale bioreactor for further upscaling. In addition to higher production capacity, critical process parameters (for example, dissolved oxygen, pH) can be monitored and controlled using a bioreactor, resulting in increased reproducibility and product quality. Assuming that the yield generated in the Erlenmeyer flask can be upscaled for all carriers, the iCELLis® 500 m^2^ seems promising.

## Supplementary Information

Below is the link to the electronic supplementary material.

Supplementary file1 (PDF 405 KB)

## Data Availability

Not applicable.
