# Supplementary material for: Upstream process optimization and micro- and macrocarrier screening for large-scale production of the oncolytic H-1 protoparvovirus
Source: Appl Microbiol Biotechnol. 2021 Nov 16;105(24):9113–24. doi: 10.1007/s00253-021-11642-y (PMC8648687; doi:10.1007/s00253-021-11642-y)
Supplement: Supplementary file 1 — Supplementary file1 (PDF 405 KB) [file 253_2021_11642_MOESM1_ESM.pdf]

Journal:

Applied Microbiology and Biotechnology

Title:

Upstream process optimization and micro- and macrocarrier screening for large-scale production of the oncolytic H-1 protoparvovirus

Authors:

Daniel Wohlfarth, Veronika Frehtman, Marcus Müller, Martin Vogel, Linh Minh Phuc Phan, Adrian Brunecker, Barbara Leuchs

Affiliation:

German Cancer Research Center, Tumor Virology, Im Neuenheimer Feld 280, 69120 Heidelberg, Germany

Corresponding author:

Barbara Leuchs

German Cancer Research Center

Tumor Virology F010

Im Neuenheimer Feld 280

69120 Heidelberg

Germany

B.Leuchs@dkfz.de; 00496221424300; Fax: 00496221424301

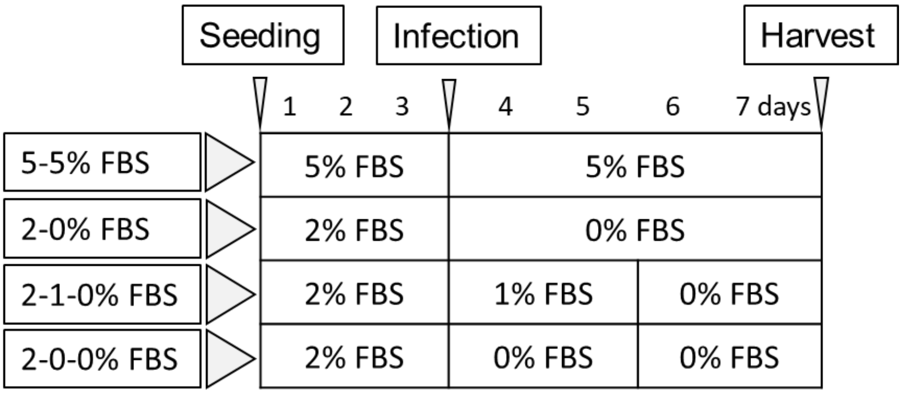

Fig. S1 Overview of process time lines and FBS content using VP-SFM™ medium utilizing nonsimultaneous seeding and infection.

Table S1: Overview of media and supplementations

Basal medium	Medium description	FBS [%]	Gln [mM]
**MEM**	Full	5	2
	W/o FBS	0	2
	Full Gln	5	5
**VP-SFM™**	Full	5	4
	Full Gln	5	6
	Cell expansion	2	4
	Infection	1	4
	W/o FBS	0	4

All media were supplemented with 100 U/ml penicillin and 100 µg/ml streptomycin

Table S2: Summary of tested parameters concerning carrier

Parameter	Parameter range
**Seeding RPM**	0-100
**Process RPM**	0-100
**Carrier density [cm²/ml]**	5-20
**Seeding density [cells/cm²]**	5E3 – 4E4
**MOI**	0.01, 0.05
**TOI [day]**	0, 3, 6, 9, 13, 16
**Bead-to-bead transfer**	(+), (-)
**Medium exchange at TOI**	50%, 100%
**Fed batch**	(+), (-)

Table S3: Summary of tested medium volumes with different vessels in carrier experiments

System	Scale	Medium volume range [ml]
**Plate**	24-well	1
**Erlenmeyer Flask**	125 ml	17.5 - 40
	1 L	150 - 300
	5 L	1500
**Spinner flask**	250 ml	100
	500 ml	100
